# Prevalence of preoperative Deep Venous Thrombosis (DVT) following elderly intertrochanteric fractures and development of a risk prediction model

**DOI:** 10.1186/s12891-022-05381-y

**Published:** 2022-05-04

**Authors:** Xiaofei Wang, Zhen Jiang, Yufu Li, Kai Gao, Yang Gao, Xiaoli He, Hongyan Zhou, Wei Zheng

**Affiliations:** 1 Department of Traditional Chinese Medicine Orthopaedics, the 3Rd Hospital of Shijiazhuang, NO.15 Tiyu South Street, Shijiazhuang, 050000 Hebei People’s Republic of China; 2 Department of Nursing, the 3Rd Hospital of Shijiazhuang, Shijiazhuang, 050000 Hebei People’s Republic of China

**Keywords:** Intertrochanteric fracture, DVT, Clinical epidemiology, Risk prediction model

## Abstract

**Background:**

This study aimed to investigate the prevalence of preoperative deep venous thrombosis (DVT) following intertrochanteric fractures in the elderly and identify the associated factors, based on which a risk prediction model was developed.

**Method:**

This was a retrospective single-center study of elderly patients presenting with intertrochanteric fractures between our institution between January 2017 and December 2020. Patients' duplex ultrasound (DUS) or venography results were retrieved to evaluate whether they had a preoperative deep venous thrombosis (DVT) of bilateral extremities, whereby patients were dichotomized. Various variables of interest on demographics, comorbidities, injury and biomarkers were extracted and their relationship between DVT were investigated. Statistically significant variables tested in multivariate logistics regression analyses were used to develop a risk prediction model.

**Results:**

There were 855 patients eligible to be included in this study, and 105 were found to have preoperative DVT, with a prevalence rate of 12.3%. Ten factors were tested as significantly different and 2 marginally significant between DVT and non-DVT groups in the univariate analyses, but only 6 demonstrated the independent effect on DVT occurrence, including history of a VTE event (OR, 4.43; 95%CI, 2.04 to 9.62), time from injury to DVT screening (OR, 1.19; 95%CI, 1.13 to 1.25), BMI (OR, 1.11; 95%CI, 1.04–1.18), peripheral vascular disease (OR, 2.66; 95%CI, 1.10 to 6.40), reduced albumin (2.35; 95%CI, 1.48 to 3.71) and D-Dimer > 1.0 mg/L(OR, 1.90; 95%CI, 1.13 to 3.20). The DVT risk model showed an AUC of 0.780 (95%CI, 0.731 to 0.829), with a sensitivity of 0.667 and a specificity of 0.777.

**Conclusion:**

Despite without a so high prevalence rate of DVT in a general population with intertrochanteric fracture, particular attention should be paid to those involved in the associated risk factors above. The risk prediction model exhibited the improved specificity, but its validity required further studies to verify.

## Introduction

Deep venous thrombosis of the lower extremities is a most common complication after hip fracture in the elderly, affecting 11.1% to 35.0% of patients despite thromboprophylaxis [1–4]. Effective screening and early diagnosis have been consistently playing the key role in prevention of subsequent adverse events, e.g. chronic venous insufficiency, secondary varicose veins, and ulcers that seriously affect patients' quality of life; and in extreme cases the lower extremity DVTs would migrate proximally to cause pulmonary embolism (PE) or even death [5]. However, limited by the physicians and equipment available and the emergent need of surgeries for hip fractures (generally within 48 h or even 24 h after injury), it is less likely or impractical to routinely perform the duplex ultrasound (DUS) or venography for detecting the potential DVT for every patient.

For purpose of early detection of DVT, identifying patient at high risk based on the related risk factors or admission laboratory biomarkers is feasible and useful, whereby risk stratification is developed and specific measures can be applied. By far, substantial efforts have been made to address this in orthopaedics or other fields, such as age-adjusting the plasma D-dimer level to improve its specificity [6–8], identifying patient-or injury-related risk factors [9–11], or some novel potential biomarkers (e.g. neutrophil or platelet to lymphocyte ratio) [12, 13]. Specific at hip fracture, importance of preventing DVT cannot be overemphasized. On one hand, patients suffering hip fracture generally have poorer systemic conditions, such as advanced age, reduced “functional reserve” organ systems and worse vascular conditions, together with sustaining hypercoagulation state and limb immobility after injury, making them more predisposed to DVT [14, 15]. On the other hand, the presence of DVT will be a provoking factor for some acute hemodynamic instability conditions, e.g. myocardial infarction or ischemic stroke or bleeding events [16], seriously complicating the surgical care. Despite the such clinical importance, existing studies on this topic are inadequate, reporting greatly variable prevalence rates of DVT and identified controversial risk factors without or limited adjustment for confounders [1, 10, 17–20], which are not necessarily applicable to populations in different settings.

Considering that intertrochanteric fracture has a higher proportion and occurs at an older age than does the femoral neck fracture, the other type of hip fracture, and further that most studies did not distinguish between both fracture types, we conducted this study. The aims were to: 1, investigate the incidence rates of preoperative DVT after intertrochanteric fractures in the elderly; 2, identify the risk factors associated with DVT; 3, based on the risk factors, to form a risk prediction model and evaluate its power.

## Materials and methods

### Inclusion and exclusion criteria

This retrospective study was performed in accordance with the Declaration of Helsinki and the study protocol was approved by the ethics committees of the 3^rd^ Hospital of Shijiazhuang. Informed consent was obtained from each participant.

Patients aged 60 years or older presenting with an isolated intertrochanteric fracture caused by a low-energy injury mechanism (fall from a standing height) diagnosed by routine radiographs and/or CT scanning between January 2017 and December 2020 were initially deemed eligible in this study. The inclusion criteria were patients having independent pre-fracture mobility, having undergone DUS or/and venography for detection of preoperative DVT, undergoing definite surgical care (osteosynthesis) and having complete data on demographics, injury, comorbidities and laboratory biomarkers of interest. The exclusions criteria were: pathological/metastatic fracture, fractures caused by high-energy injury mechanism, open fracture, bilateral hip fractures, multiple trauma or concurrent fractures of other sites, malignancy, patients having undergone thromboembolic drug prophylaxis before DVT screening, thrombotic event having occurred during last 30 days prior to index fracture, current use of corticosteroids or anticoagulants within 3 months of injury, delay to surgery exceeding 21 days and incomplete data of interest.

### Diagnosis and management of DVT

DVT was diagnosed in accordance with the specific guideline proposed by the Chinese Medical Association (3^rd^ edition) (16). Based on our institution policy, for suspected or high-risk DVT following major extremity trauma (e.g. hip fracture) or before/after the major orthopaedic surgeries, detection of DVT via DUS or/and venography of the lower extremities is mandatory to reduce the occurrence of serious adverse events.

Physical prophylaxis such as elevation of injured extremity, quadriceps strength exercise and ankle pump practices are routinely applied for each patient once admission. Based on the DVT detection results, prophylactic or therapeutic chemical thromboprophylaxis was administered, including subcutaneous injection of low-molecular-weight heparin (LMWH), direct oral anticoagulants (factor Xa inhibitors, e.g. rivaroxaban, apixaban, edoxaban).

### Data collection

Patients' electronic medical records were inquired for exaction of relevant data, including demographics (age, sex, body weight and height, residence place, occupation and education level), comorbidities or disorders (hypertension, diabetes mellitus, chronic heart disease, history of cerebrovascular disease, respiratory disease, liver disease (hepatitis or cirrhosis), renal insufficiency, peripheral vascular disease and American Society of Anesthesiologists (ASA) classification, history of VTE), injury-related data (affected side, time from injury to admission and to DVT screening and fracture classification based on AO classification system). The laboratory biomarker results at admission, generally the first one, were extracted and recorded for white blood cell (WBC), neutrophil, lymphocyte, red blood cell (RBC) and platelet, serum albumin, triglyceride, total cholesterol, sodium, hemoglobin, hematocrit, platelet distribution width (PDW), red cell distribution width (RDW); hypersensitive C-reactive protein (HCRP); fasting blood glucose (FBG), D-dimer, prothrombin time (PT), activated partial thromboplastin time (APTT), thrombin time (TT), Fibrinogen Degradation Products (FDP) and fibrinogen). For further exploration of the novel biomarkers potentially relating to DVT, we also included neutrophil to lymphocyte ratio (NLR) and platelet to lymphocyte ratio (PLR), which both were repeatedly investigated in literature and in some studies demonstrated association to a certain extent [21, 22].

### Statistical analysis

Distributions of continuous variables were evaluated using the Shapiro-Wilkes test. Normally distributed continuous variables were presented as mean ± standard deviation (SD) and between-group difference was examined using Student-t test; while non-normally distributed variables were presented as median and interquartile range (IQR) and examined by Whitney-U test Mann analyzed using Student-t test for normally distributed data, and Mann Whitney-U test for the non-normally distributed data. Categorical variables were presented as a number and a percentage, and between-group differences were examined by Chi-square test or Fisher exact test, as appropriate.

For laboratory indexes and NLR or PLR, we did not apply the fixed and traditionally well-established reference range to classify them, because most of them were age- or trauma-dependent [6, 7, 23, 24], especially in such setting of a major trauma in aged patients. Instead, we constructed receiver operating was maximized. The area under the ROC (AUC) was used to evaluate the ability to distinguish between DVT and non-DVT [25], with variables with statistical significance (*p* < 0.05) considered to be dichotomized for a univariate analysis.

Then, variables including biomarkers tested with ≤ 0.10 in the univariate analysis were included in the multivariate logistics regression model to determine the independent risk factors. In this process, stepwise backward method was used and variables with *p* ≤ 0.10 were retained in the final model. The model fit was evaluated by the Hosmer–lemeshow (H–L) goodness of fit test, with statistics > 0.05 indicating an acceptable result. The correlation magnitude of a factor with DVT was represented by the odds ratio (OR) and 95% confidential interval (95%CI). Based on multivariate findings, the combination prediction model was established and ROC curve was constructed to determine its predictive ability [25].

The statistical significance was set as *P* < 0.05 and all the analyses were performed using SPSS24.0 (IBM corporation, New York, USA).

## Results

Initially, the crude strategy retrieved 1787 cases of intertrochanteric fractures. Nine hundred and thirty-two cases were excluded, due to younger age (*n* = 229), middle- or high-impact mechanism (*n* = 126), pre-fracture dependent mobility (*n* = 63), pathological/metastatic fracture (*n* = 19), open fracture (*n* = 9), bilateral hip fractures (*n* = 12, cases of fracture), multiple trauma or concurrent fractures of other sites (*n* = 39), conservative treatment (*n* = 31), malignancy (*n* = 22), thromboembolic drug prophylaxis before DVT screening (*n* = 146), thrombotic event having occurred during last 30 days prior to index fracture (*n* = 37), corticosteroids or anticoagulants taken within 3 months of injury (*n* = 24), delay to surgery exceeding 21 days (*n* = 91), or missing data on variables (*n* = 84).

Finally, there were 855 patients included 312 males and 543 females, with a mean age of 77.2 ± 8.5 years (range, 60 to 103 years; IQR, 71 to 84 years). There were 105 patients diagnosed with preoperative DVTs, indicating an incidence rate of 12.3% (95%CI, 10.1% to 14.5%). The time from injury to admission was 1.9 ± 3.3 days, to DVT screening was 4.1 ± 3.7 days. The time from admission to operation was 3.8 ± 2.4 days. Stratified by factors, the incidence rate of preoperative DVT was 14.4% (45/312) for males versus 11.0% (60/543) for females (*p* = 0.148), 16.2% in those aged 60–69 years versus 13.3% in 70–79, 9.0% in 80–89 and 13.7% in ≥ 90 years (*p* = 0.099), 5.0% (10/201) in those undergoing DVT examination within 2 days after injury, 8.4% (38/450) in 3–6 days, and 27.9% (54/204) after 7 days (*P* < 0.001).

Eight biomarkers or indexes were found to have a significant ability to distinguish between DVT and non-DVT, including D-Dimer, TT, PT, albumin, RBC, HCT, platelet and PLR, with respective optimal cut-off value of 1.0 mg/L, 15.5 s, 11.5 s, 32.5 g/L, 2.7*10^12^/L, 34%, 214*10^9^/L and 174, based on which they were dichotomized (Table 1).

**Table 1:** The AUC used to evaluate the ability to distinguish between DVT and non-DVT patients, and to determine the optimal cut-off value for some variables with significance (*p* < 0.05)

Variable	AUC	95%CI		SE	P	Optimal Cut-off value
		**Lower limit**	**Upper limit**			
**D-dimer**	0.582	0.525	0.640	0.029	*0.006	1.0 mg/L
**TT**	0.563	0.510	0.616	0.027	*0.035	15.5 s
**PT**	0.576	0.521	0.631	0.028	*0.012	11.5 s
**ALB**	0.614	0.559	0.668	0.028	* < 0.001	32.5 g/L
**RBC**	0.559	0.503	0.615	0.028	*0.050	2.7*10^12^/L
**HCT**	0.559	0.505	0.614	0.028	*0.049	34%
**Platelet**	0.573	0.515	0.634	0.030	0.014	214*10^9^/L
**PLR**	0.587	0.529	0.645	0.029	*0.004	174
**TC**	0.575	0.516	0.634	0.030	0.012	3.45
**TG**	0.516	0.458	0.574	0.030	0.593	NA
**WBC**	0.494	0.436	0.553	0.030	0.848	NA
**Neutrophil**	0.499	0.441	0.558	0.030	0.985	NA
**FIB**	0.514	0.456	0.572	0.030	0.636	NA
**FDP**	0.540	0.485	0.595	0.028	0.184	NA
**APTT**	0.534	0.477	0.591	0.029	0.263	NA
**PDW**	0.545	0.486	0.603	0.030	0.136	NA
**RDW**	0.521	0.460	0.581	0.031	0.495	NA
**HCRP**	0.517	0.461	0.573	0.029	0.573	NA
**Sodium**	0.552	0.489	0.614	0.032	0.087	NA
**FBG**	0.529	0.472	0.585	0.029	0.341	NA
**NLR**	0.518	0.458	0.578	0.031	0.542	NA
**HGB**	0.558	0.502	0.613	0.028	0.054	NA

***Abbreviations**** TT* thrombin time, *PT* prothrombin time, *PTT*, activated partial thromboplastin time, *ALB* albumin, *RBC* red blood cell, *HCT* hematocrit, *HGB* hemoglobin, *PLR* platelet to lymphocyte ratio, *NLR* neutrophile to lymphocyte ratio, *FIB* fibrinogen, *WBC* white blood cell, *PDW* platelet distribution width, *RDW* red cell distribution width, *HCRP* Hypersensitive C-reactive protein, *FBG* fasting blood glucose, *TG* triglyceride, *TC* total cholesterol, *AUC* area under curve, *CI* confidence interval, *SE* standard error, *ND* not available

In the univariate analyses, significant difference was found between DVT and non-DVT groups in term of BMI (24.4 ± 3.7 kg/m^2^ vs 23.2 ± 3.4 kg/m^2^), prevalence rate of peripheral vascular disease (8.6% vs 2.9%) and a history of a VTE event (13.3% vs 4.5%), time from injury to DVT screening (7.7 ± 4.5d vs 3.6 ± 3.3d) platelet count > 214*109/L (53.3% vs 38.8%), HCT < 34% (80.0% vs 66.9%), D-Dimer level > 1.0 mg/L (78.1% vs 62.0%), PLR > 174 (67.6% vs 51.7%), albumin level < 32.5 g/L (62.9% vs 41.3%) and TC < 3.45 mmol/L (49.5% vs 35.1%). Patients with DVT had a tendency toward higher prevalence of PT < 11.5 s (31.4% vs 23.2%) and ASA III-IV (57.1% vs 47.6%), although not approaching to significance (*P* = 0.065 and *P* = 0.067) (Table 2).

**Table 2:** Univariate analysis of factors associated with preoperative deep venous thrombosis (DVT)

Variables	Number (%) of patients without DVT (*n* = 750)	Number (%) of patients with DVT (*n* = 105)	*P*
**Gender (males)**	267 (35.6)	45 (42.9)	0.148
**Age**	77.4 ± 8.4	76.2 ± 8.9	0.184
**Body mass index (BMI)**	23.2 ± 3.4	24.4 ± 3.7	0.002
**Hypertension**	434 (57.9)	60 (57.1)	0.888
**Diabetes mellitus**	140 (18.7)	17 (16.2)	0.539
**Heart disease**	242 (32.3)	32 (30.5)	0.713
**Cerebrovascular disease**	160 (21.3)	18 (17.1)	0.322
**Pulmonary disease**	77(10.3)	9 (8.6)	0.589
**Liver disease**	37 (4.9)	2 (1.9)	0.164
**Renal insufficiency**	28 (3.7)	3 (2.9)	0.653
**Peripheral vascular disease**	22 (2.9)	9 (8.6)	0.004
**History of allergy**	155 (20.7)	19 (18.1)	0.540
**History of surgery**	209 (27.9)	30 (28.6)	0.880
**History of VTE event**	26 (3.5)	14 (13.3)	< 0.001
**Time from injury to DVT screening**	3.6 ± 3.3	7.7 ± 4.5	< 0.001
**Fracture classification (based on AO classification system)**			0.445
** A1**	238 (31.7)	31 (29.5)	
** A2**	406 (54.1)	63 (60.0)	
** A3**	106 (14.1)	11 (10.5)	
**ASA score**			0.067
I-II	393 (52.4)	45 (42.9)	
III-IV	357 (47.6)	60 (57.1)	
PLT (> 214*10^**9**^**/L)**	291 (38.8)	56 (53.3)	0.005
**HCT (**< 34.0%**)**	502 (66.9)	84 (80.0)	0.007
**D-dimer (> 1.0 mg/L)**	465 (62.0)	82 (78.1)	0.001
**PT < 11.5 s**	174 (23.2)	33 (31.4)	0.065
**PLR > 174**	388 (51.7)	71 (67.6)	0.002
**ALB (< 32.5 g/L)**	310 (41.3)	66 (62.9)	< 0.001
**TT(> 15.5 s)**	524 (69.9)	72 (68.6)	0.787
**TC (< 3.7 mmol/L)**	263 (35.1)	52 (49.5)	0.004

**Fig. 1:**
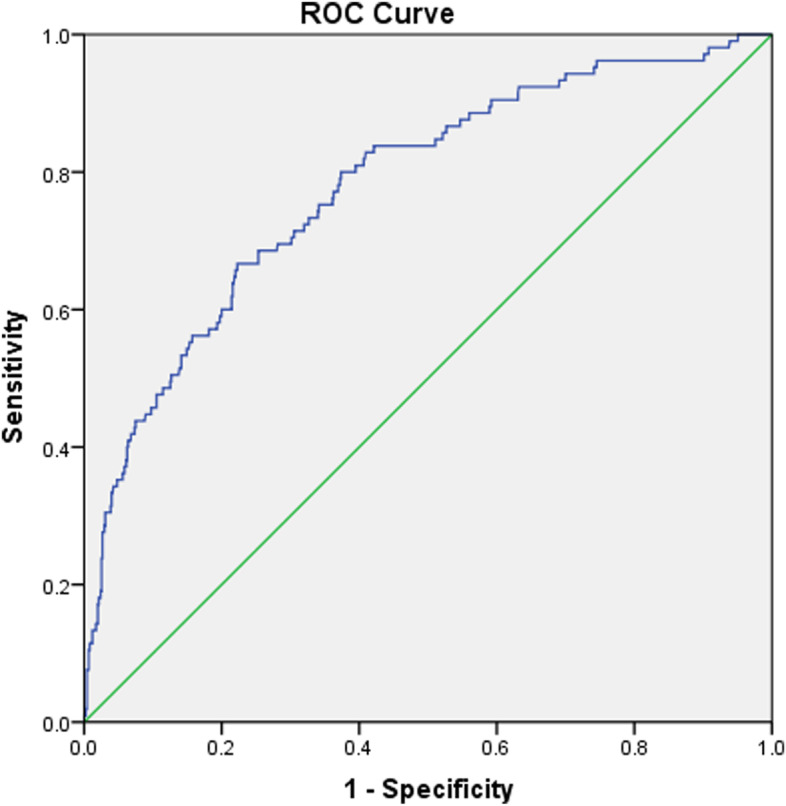
The ROC curve for the developed risk prediction model, which had an AUC of 0.780 (95%CI, 0.731 to 0.829), with a sensitivity of 0.667 and a specificity of 0.777, when Youden index was maximized

In the multivariate analyses, all the 12 above-mentioned variables including PT and ASA classification were included, and the results showed that history of a VTE event (OR, 4.43; 95%CI, 2.04 to 9.62), time from injury to DVT screening in each day increment (OR, 1.19; 95%CI, 1.13 to 1.25), BMI in each kg/m^2^ increment (OR, 1.11; 95%CI, 1.04–1.18), peripheral vascular disease (OR, 2.66; 95%CI, 1.10 to 6.40), albumin < 32.5 g/L (OR, 2.35; 95%CI, 1.48 to 3.71) and D-Dimer > 1.0 mg/L (OR, 1.90; 95%CI, 1.13 to 3.20) (Table 3**)**. Based on these variables, the DVT risk model was formed, showing an AUC of 0.780 (95%CI, 0.731 to 0.829), with a sensitivity of 0.667 and a specificity of 0.777, when Youden index was maximized (Fig. 1).

**Table 3:** Multivariate analysis of factors associated with preoperative DVT following an intertrochanteric fracture

Variables	OR	95%CI		*P*
		**lower limit**	**upper limit**	
History of VTE	4.43	2.04	9.62	< 0.001
Peripheral vascular disease	2.66	1.10	6.40	0.029
BMI (each kg/m^2^ increment)	1.11	1.04	1.18	0.001
Delay to DVT examination (each day increment)	1.19	1.13	1.25	< 0.001
Albumin (< 32.5 g/L)	2.35	1.48	3.71	< 0.001
D-Dimer > 1.0 mg/L	1.90	1.13	3.20	0.016

The H–L test showed the adequate goodness of fit (X^2^ = 7.537, *P* = 0.480, Nagelkerke R^2^ = 0.218) for the final multivariate model.

## Discussion

Prevention and management of perioperative complications is a consistent subject in practice and research in elderly hip fracture, especially for those prevalent and further catastrophic complications. In this study, we used the sample from a tertiary referral trauma center and found preoperative DVT had prevalence of 12.3% in elderly patients with a hip fracture, and identified 6 independent factors associated with DVT, including history of a VTE event, time from injury to DVT screening, BMI, peripheral vascular disease, lower albumin level and elevated D-Dimer. The combination risk prediction model, developed based on these findings, showed a favorable performance in distinguishing between DVTs and non-DVTs, with an AUC of 0.780.

We reported a relatively lower prevalence rate of preoperative DVT following elderly intertrochanteric fractures, compared to those in literature. Zuo et al. [18] reported a 20.1% rate of admission DVT diagnosed by DUS in their retrospective analysis of 578 intertrochanteric fractures in patients aged 60 years or older. Fei et al. [20], in their retrospective study of 218 patients aged 16 years or older with intertrochanteric fractures, found the preoperative DVT diagnosed by DUS was 37.6%, about 3 times as ours. Bengoa et al. [26] investigated the relationship between DVT prevalence and the delay to admission, and found 17.6% of prevalence in patients admitted ≥ 48 h after a hip fracture, which was consistent with ours (14.5%, 95/654). In the other studies with different settings related to patients or fracture types (including or limited to femoral neck fracture), investigators reported the variable prevalence rates, from 11.% to 35.0% [1–3]. The great variation in DVT prevalence reflected the differences in race, patient characteristics, study design, DVT screening methods and the policies on DVT prophylaxis and hip fracture care, although it was almost impossible in a perfectly homogeneous population. In our study we used the more stringent criteria that only patients without DVT chemoprophylaxis before DVT examination could be included. In addition, for purpose of continuous exploration of potentially new factors, we excluded those with a well-established factor, e.g. recent incident thrombotic events (i.e., within 1 month before index fracture) or pre-fracture mobility dependence, because as per institutional policy, patients with these conditions would be classified as high-risk group and be given targeted therapeutic intervention (double-dose LMWH, compared to single-dose for prophylactic intervention) as a matter of priority. These are likely contributing to our examined low prevalence rate of DVT.

Among the 6 independent factors identified, most were repeatedly investigated in literature, such as delay to admission or DVT screening [10, 17–19], higher BMI or obesity [18, 19], peripheral vascular disease [19], reduced albumin [18] and elevated D-Dimer level [1, 10, 17–19]. The relatively prolonged time from injury to DVT examination (mean, 4.1 days) must be explain. In fact, in most tertiary referral hospitals in China, including ours, it is not easy to follow the early-surgery-within-48-h recommendation, and the prolonged “wait” is primarily involves the time from injury to admission. This institution is an 800-beds-setting orthopaedics-specialized hospital, covering over 10 million inhabitants in Shijiazhuang City. Despite that, a substantial proportion of patients will wait for some days before admitting, due to the relatively inadequate beds; and during COVID-19, this situation is even more deteriorating due to the strict hospitalization policy regarding compulsory and within-48 h nucleic acid testing negative result. In almost all previous studies, history of a VTE was excluded due to its potential strong effect on the secondary VTE, which subjected to be provoked by an acute trauma (e.g. fracture or bleeding event) or a medically unstable status (e.g. cerebral or cardiac ischemia). In our study, we found the strongest magnitude of association of history of VTE (OR, 4.43) with the DVT, underscoring the importance of classifying patients with a history of VTE as high-risk population in elderly intertrochanteric fracture practice, regardless of presence of other risk factors.

Plasma D-dimer level is a typical laboratory biomarker for diagnosis of DVT or PE, but its diagnostic value in some specific groups of patients remains in controversy. Substantial evidences have demonstrated the age-dependence of D-dimer concentration, and the conventional cut-off value (0.5 mg/L) is scarcely able to provide a discrimination between VTEs and non-VTEs in the elderly patients. According to a systematic review, the specificity of D-dimer test with a traditional cut-off value was 49% to 67% in patients aged < 50 years, but between 0 and 18% in those ≥ 80 years [27]. Given that most intertrochanteric fracture occurred at an advanced age, mean of 77 years in this study, we re-defined cut-off value of D-dimer as 1.0 mg/L and demonstrated its acceptable power (sensitivity, 0.781; specificity, 0.380). Future studies on investigation of age-adjusted D-dimer value on DVT at a specific group, e.g. hip fracture patients, are warranted to refine its value.

Improvement of the specificity in diagnosis of VTE have been an increasingly important subject via various methods, such as adjusting the age-related D-dimer coefficient, combination diagnostic test, or development of risk models based on the identified factors, to reduce the unnecessary diagnostic imaging investigation [27–29]. However, specific at hip fractures generally requiring emergent surgeries, there were few effective and practical methods and related studies were inadequate. In this study, we developed this risk prediction model based on the 6 risk factors identified, which exhibited a moderate sensitivity of 0.667, but importantly the relatively high specificity of 0.777. This may compensate for the low specificity of D-dimer in traditional (0.5 mg/L, about 10%-30%) [30, 31] or current cut-off (1.0 mg/L, 38%) in the elderly patients, contributing to safely ruling out DVT in a substantial proportion of hip fracture patients. Despite this, given the potential serious consequence of the DVT and that surgeons do not always get the chance to reconsider a missed diagnosis in such major trauma requiring emergence surgeries, the subsequent prospective studies with large sample are needed to verify the effectiveness and safety of this risk prediction model.

The strengths of this study were the strict and exclusion criteria, the large sample of participants, redefining the optimal cut-offs for some DVT-closely-related biomarkers and development of a risk prediction model with high specificity for a specific population of elderly intertrochanteric fracture patients. However, the limitations should be noted. First, the retrospective design introduced the bias in accuracy in data collection, especially on comorbidities that were self-reported by patients or relatives. It is possible that some already existing comorbidities are not identified or diagnosed, and therefore underreported. Second, this was a single-center study in a tertiary referral trauma center, which biased the patient selection because patients admitted had a severer fracture or more complex medical conditions. The generalizability of the findings is limited; especially, the results including the presented DVT rate are more applicable to Chinese healthcare system. Third, for outpatients or inpatients, Wells or Caprini score are most important tool for risk-classifying patients for the risk of DVT. However, due to absence of many variables, such as inflammatory bowel disease, positive for lupus anticoagulant Heparin-induced thrombocytopenia, central venous access or serum homocysteine et al., and exclusion of some well-known factors like pre-fracture not independent mobility, VTE history, and recent use of corticosteroids or anticoagulants, we could not obtain the corresponding score, which might have lowered its practicability. Fourth, we collected laboratory data at admission for analysis, most of which, however, were variable in time. In our study, the wide span of admission would have affected the results, despite we have adjusted for this variable. Fitth, as every multivariate analysis, there remains residual confounding effects from unknown or unmeasured factors. Fifth, this is first attempt to develop a DVT risk prediction model in such a population, and so its validity necessitates further well-designed studies to verify.

## Conclusions

In conclusion, we found a relatively low incidence rate of preoperative DVT in a group of elderly intertrochanteric fracture patients, and identified 6 factors including history of a VTE event, time from injury to DVT screening, BMI, peripheral vascular disease, lower albumin level and elevated D-Dimer as independently associated with DVT. By far as we know, this is first to conduct so detailed and extensive investigation of biologic indictors in relation to DVT. The risk prediction model based on these factors exhibited the improved power in predicting the DVT following intertrochanteric fracture, and may facilitate the management of such injury in already frail elderly patients for reduction of unexpected adverse events.

## Data Availability

All the data will be available upon motivated request to the corresponding author of the present paper.

## Abbreviations

DVT: Deep venous thrombosis
TT: Thrombin time
PT: Prothrombin time
APTT: Activated partial thromboplastin time
ALB: Albumin
RBC: Red blood cell
HCT: Hematocrit
HGB: Hemoglobin
PLR: Platelet to lymphocyte ratio
NLR: Neutrophile to lymphocyte ratio
FIB: Fibrinogen
WBC: White blood cell
PDW: Platelet distribution width
RDW: Red cell distribution width
HCRP: Hypersensitive C-reactive protein
FBG: Fasting blood glucose
TG: Triglyceride
TC: Total cholesterol
AUC: Area under curve
CI: Confidence interval
SE: Standard error
OR: Odd ratio
VTE: Venous thromboembolism
